# Inhibition of MC38 colon cancer growth by multicomponent chemoimmunotherapy with anti-IL-10R antibodies, HES-MTX nanoconjugate, depends on application of IL-12, IL-15 or IL-18 secreting dendritic cell vaccines

**DOI:** 10.3389/fimmu.2023.1212606

**Published:** 2023-07-20

**Authors:** Katarzyna Węgierek-Ciura, Jagoda Mierzejewska, Agnieszka Szczygieł, Joanna Rossowska, Anna Wróblewska, Marta Świtalska, Tomasz M. Goszczyński, Bożena Szermer-Olearnik, Elżbieta Pajtasz-Piasecka

**Affiliations:** Hirszfeld Institute of Immunology and Experimental Therapy, Polish Academy of Sciences, Wrocław, Poland

**Keywords:** dendritic cells, interleukin 12, interleukin 15, interleukin 18, anti-IL-10R antibodies, nanoconjugate, methotrexate, colon carcinoma

## Abstract

**Background:**

The tumor microenvironment (TME) provides a conducive environment for the growth and survival of tumors. Negative factors present in TME, such as IL-10, may limit the effectiveness of cellular vaccines based on dendritic cells, therefore, it is important to control its effect. The influence of IL-10 on immune cells can be abolished e.g., by using antibodies against the receptor for this cytokine - anti-IL-10R. Furthermore, the anticancer activity of cellular vaccines can be enhanced by modifying them to produce proinflammatory cytokines, such as IL-12, IL-15 or IL-18. Additionally, an immunomodulatory dose of methotrexate and hydroxyethyl starch (HES-MTX) nanoconjugate may stimulate effector immune cells and eliminate regulatory T cells, which should enhance the antitumor action of immunotherapy based on DC vaccines. The main aim of our study was to determine whether the HES-MTX administered before immunotherapy with anti-IL-10R antibodies would change the effect of vaccines based on dendritic cells overproducing IL-12, IL-15, or IL-18.

**Methods:**

The activity of modified DCs was checked in two therapeutic protocols - immunotherapy with the addition of anti-IL10R antibodies and chemoimmunotherapy with HES-MTX and anti-IL10R antibodies. The inhibition of tumor growth and the effectiveness of the therapy in inducing a specific antitumor response were determined by analyzing lymphoid and myeloid cell populations in tumor nodules, and the activity of restimulated splenocytes.

**Results and conclusions:**

Using the HES-MTX nanoconjugate before immunotherapy based on multiple administrations of anti-IL-10R antibodies and cellular vaccines capable of overproducing proinflammatory cytokines IL-12, IL-15 or IL-18 created optimal conditions for the effective action of these vaccines in murine colon carcinoma MC38 model. The applied chemoimmunotherapy caused the highest inhibition of tumor growth in the group receiving DC/IL-15/IL-15Rα/TAg + DC/IL-18/TAg at the level of 72.4%. The use of cellular vaccines resulted in cytotoxic activity increase in both immuno- or chemoimmunotherapy. However, the greatest potential was observed both in tumor tissue and splenocytes obtained from mice receiving two- or three-component vaccines in the course of combined application. Thus, the designed treatment schedule may be promising in anticancer therapy.

## 1 Introduction

According to the World Health Organization, colon cancer was the second leading cause of cancer-related deaths worldwide in 2020 (1). Growing evidence has demonstrated that the fate of tumor progression is highly correlated with the presence of a specific tumor microenvironment (TME) created by tumor cells, extracellular matrix, stromal cells, as well as various types of infiltrating immune cells (2), which activity is modified by TME. For example, macrophages originating from circulating monocytes, under the influence of tumor-derived factors, can become tumor-associated macrophages (TAMs) regarded as the leading producers of immunosuppressive cytokines such as IL-10 and TGF-β (3). TAMs are able to present antigens in the MHC II-associated pathway, nevertheless, in hypoxia or under the influence of IL-10, the MHC II surface expression decreases, causing tumor progression (4–6). Myeloid-derived suppressor cells (MDSCs), other populations of myeloid cells which produce IL-10, under hypoxia conditions are able to transform into TAMs increasing the size of this population (7). TME contributes to a change in the infiltration of both T and natural killer (NK) cells. However, depending on the type of environmental factors or interaction with MDSCs, part of effector CD4^+^ cells can convert into regulatory T cells (Tregs), which are involved in the suppression of arising antitumor activity by secretion of both TGF-β and IL-10, and their increased frequency is associated with poorer cancer patients’ prognoses (8, 9).

The use of conventional treatment with various chemotherapeutics aimed at the elimination of cancer cells is still unsatisfactory, especially in the context of severe systemic side effects. Hence there is a requirement for new, safe and effective anticancer therapies based on cytostatics, for example, using novel drug delivery systems. Such an approach is the use of nanoconjugate of methotrexate and hydroxyethyl starch (HES-MTX), which was obtained by covalent coupling of two well-known therapeutic compounds – methotrexate (MTX) and hydroxyethyl starch (HES) (10). MTX represents one of the oldest antifolate chemotherapeutics, commonly used in autoimmune diseases and in anticancer therapy of solid tumors and hematologic malignancies (11), whereas HES as an amylopectin-based modified polymer is applied in medicine as colloidal plasma volume expander. The HES-MTX nanoconjugate provides a prolonged half-time in plasma compared to the free form of MTX, which further improves the distribution of the nanoconjugate in the body and effective drug release in the target tissue (10). The effect of conjugation of a low-molecular-weight drug with a high-molecular-weight carrier provides an optimal hydrodynamic diameter of the obtained HES-MTX nanoconjugate. This enables more efficient accumulation in tumor tissue, mainly through enhanced vascular permeability and retention effect (EPR), which was discussed in more detail in our previous publication (12). Moreover, the anticancer activity of HES-MTX has been confirmed in murine and human leukemia models (10), as well in the murine colon carcinoma MC38 model (12, 13).

Secreted by many leukocyte populations, IL-10 is one of the factors present in TME, contributing to the suppression of immune response (14, 15). This cytokine is involved in inhibiting antigen presentation, affects the differentiation and maturation of DCs and impairs their migration to secondary lymphoid organs. Moreover, IL-10 reduces the local antigen-specific response of CD8^+^ cells and suppresses IL-12 secretion by DCs (16, 17). Therefore, the manipulation of IL-10 levels is a key step in controlling tumors, especially in the advanced stages of the disease (18). This can be achieved by local or systemic blockade of IL-10 activity, e.g. by administration of shRNA against IL-10 and antibodies neutralizing IL-10 or IL-10 receptor (19–24). The temporary blockade of the IL-10-specific receptor (IL-10R) with anti-IL-10R antibodies reduces the sensitivity of immune cells to IL-10, which prevents the transformation of effector cells into suppressor cells and prepares optimal conditions for triggering an efficient anticancer response.

The immune cells’ responsiveness can be also restored using dendritic cell-based vaccines. Effective therapeutic vaccines should be able to prime naïve T cells, but most importantly, induce the transition of existing memory T cells to effector CD8^+^ cells. To effectively stimulate T lymphocytes, dendritic cells must present tumor antigens *via* MHC class I and II molecules, express costimulatory ligands, and inflammatory mediators such as IL-12 or type I interferons (IFNs) (25). Accumulating evidence indicates the possibility of using such vaccines to support conventional therapies. After using combined therapy, it is possible to obtain a synergistic effect, especially when DCs have not only been subjected to antigenic activation but also genetic modifications. The efficacy of the combined therapy may therefore rely on the use of genetically modified DCs secreting cytokines (e.g. IL-12, IL-15) enhancing the efficacy of the activated DCs to induce an antitumor response (26). Proinflammatory cytokines such as IL-12, IL-15 or IL-18 can additionally stimulate the proliferation and activity of the immune system cells.

Interleukin 12 regulates inflammation by harnessing effector mechanisms of both innate and acquired immunity (27). Most IL-12-induced effects are mediated by the IFN-γ secretion and promotion of the Th1 T helper cell differentiation (28). The effect of IL-12 on the antitumor response has been observed in preclinical models, but it has not been implemented in clinical practice due to substantial side effects after systemic administration. Thus, the delivery of IL-12 for therapeutic purposes focuses on its direct application to the tumor site (29). In turn, IL-15 stimulates the activation, proliferation, survival, and cytotoxicity of CD8^+^ cells and memory phenotype CD8^+^ cells. Like IL-2, IL-15 could be used to proliferate and maintain NK cells and is critical for the functional maturation of both macrophages and DCs. However, unlike IL-2, IL-15 does not lead to activation-induced T cell death, proliferation enhancement, or differentiation of immunosuppressive CD4^+^ Treg cells (30, 31). Multiple murine immunotherapy trials in different models have revealed that IL-15 might be more effective than IL-2 in anticancer therapy (32). Still, when administered as monotherapy it has appeared to be ineffective, needing the combinate of application with other anticancer agents (31). Interleukin 18 was known as an IFN-γ inducing factor due to its function in IFN-γ enhancement. This leads to the polarization of CD4^+^ cells towards the helper T cell type 1 (Th1) phenotype when costimulated with IL-12 or IL-15. Importantly, without IL-12 or IL-15, IL-18 does not induce IFN-γ production but plays an important role in differentiating naive T cells into Th2 cells producing IL-13 and IL-4. Using IL-18 as an adjuvant combination with other cytokines, such as IFN-α, IL-15, IL-12, and IL-2, it can also promote interaction between DCs and T cells, and the activation and proliferation of T cells in colorectal cancer patients (33, 34).

The main purpose of our study was to determine the effect of immunotherapy alone based on multiple administrations of cellular vaccines and anti-IL-10R antibodies and evaluate the efficacy of the therapy, which was preceded by a single administration of the HES-MTX nanoconjugate in an immunomodulatory dose. Based on tumor growth inhibition and changes in local and systemic immune responses, we have shown that using the HES-MTX nanoconjugate creates a suitable environment for the effective action of cell-based vaccines. This was especially evident in the groups receiving DC vaccines based on mixtures of two or three transductants, although the concentration of a single cytokine released was lower than when a single transductant was used. Such therapy resulted in a clear inhibition of tumor growth and a decrease in the population of TAM and Treg cells with a suppressor effect. There was also observed an increase in the infiltration of CD4^+^, CD8^+^ cells and NK cells into tumor tissue, especially after chemoimmunotherapy harnessing anti-IL-10R antibodies and mixtures of dendritic cells.

## 2 Materials and methods

### 2.1 Cell lines

The *in vivo* growing MC38 murine colon carcinoma from the Tumor Bank of the Radiobiological Institute TNO (Rijswijk, The Netherlands) was adapted to *in vitro* conditions as described by Pajtasz−Piasecka et al. (35). The culture of MC38/0 (named here MC38) cells was maintained in RPMI−1640 (Gibco) supplemented with 100 U/ml penicillin, 100 mg/ml streptomycin, 0.5% sodium pyruvate, 0.05 mM 2−mercaptoethanol (named here RPMI) and 5% fetal bovine serum (FBS; all reagents from Sigma−Aldrich). Lenti-X 293T cell line (Clontech) was maintained in high-glucose Dulbecco’s Modified Eagle Medium (Gibco) supplemented with 100 U/ml penicillin, 100 mg/ml streptomycin, 1 mM sodium pyruvate and 10% FBS. Cultures were carried out under standard conditions (5% CO_2_, 37°C).

### 2.2 Tumor cell lysate preparation

The MC38 colon cancer cells were harvested and resuspended at a density of 5×10^6^ cells/mL in RPMI-1640 (Gibco) supplemented with 10% FBS (Sigma-Aldrich). All cells were subjected to 5 cycles of freezing in liquid nitrogen and quick thawing at 37°C. The cell suspension was then sonicated for 90 min. Tumor antigens (TAg) were used to stimulate dendritic cells.

### 2.3 Production of lentiviral vectors

Lentiviral vectors (LVs) were produced using a third-generation lentiviral system, which consisted of auxiliary vectors: pMDLg/pRRE, pRSV Rev, pMD2.G [the plasmids were a gift from Didier Trono (Addgene plasmids #12251, 12253, 12259)] and expression vector. The expression plasmids encoded the cytokine genes: *il12*, *il15/il15ra* or *il18*. In the vector carrying the *il15* gene, the gene sequence of this cytokine was preceded by a signal sequence facilitating the release of the protein from the cell. In addition, after the gene encoding the cytokine and the linker, there was a gene sequence encoding the alpha subunit of the IL-15 receptor, in order to delay the process of intracellular degradation of this protein. A control vector was also created to check the effect of lentiviral transduction on dendritic cells. All expression vectors were purchased from VectorBuilder. Lentiviral vectors were produced by Lenti-X 293T cells (ClonTech) co-transfected with the above-mentioned plasmids and cultured for 48 hours. The supernatant containing lentiviral vectors was collected and concentrated by precipitation using PEG 6000 (Sigma-Aldrich). The pellet containing the lentiviral vectors was suspended in PBS and stored at -80°C. The procedure of lentiviral vectors production was described in our previous article (36). The titer of the lentiviral vector was determined by serial dilution method using MC38 cells and flow cytometry analysis.

### 2.4 Animals

Female C57BL/6 mice were obtained from the Center for Experimental Medicine of the Medical University of Białystok, Poland. All experiments were performed in accordance with Directive 2010/63/EU of the European Parliament and of the Council on the protection of animals used for scientific purposes and were approved by the Local Ethic Committee for Experiments with the Use of Laboratory Animals, Wrocław, Poland (authorization number 077/2019). After the experiments, mice were sacrificed by cervical dislocation.

### 2.5 Generation of cellular vaccines based on transduced dendritic cells

Dendritic cells were generated from bone marrow isolated from femurs and tibias of healthy female C57BL/6 mice according to the protocol described in our previous publication (37). Cells (named here DCs) were cultured in RPMI-1640 (Gibco) supplemented with 10% FBS (Sigma-Aldrich) in the presence of recombinant murine (rm)GM−CSF (ImmunoTools, 40 ng/ml) and rmIL−4 (ImmunoTools, 10 ng/ml). After 7 days of culture, loosely attached immature DCs were transduced with LVs (with the assumption: 4 viral infectious particles per 1 dendritic cell) in the presence of 8 µg/ml polybrene (Sigma−Aldrich). After 4 hours DCs, were stimulated with tumor antigen lysates (TAg, 10% v/v). Mature dendritic cells obtained on day 8 of DC culture were collected and applied to *in vitro* DC characteristics or used as cellular vaccines in *in vivo* experiments.

### 2.6 Phenotype characteristic of transduced DCs

In order to estimate the influence of LV transduction on the differentiation level of DCs, the phenotype characteristic was performed on 8 and 10 days of DC culture by flow cytometry. Cells were labeled with a cocktail of monoclonal antibodies conjugated with fluorochromes: anti-CD11c Brilliant Violet 650 (clone N418), CD80 PerCP-Cy5.5 (clone 16-10A1), CD86 PE-Cy7 (clone GL-1), MHC II APC-Fire 750 (clone M5/114.15.2) (all from BioLegend) and CD40 Brilliant Violet 605 (clone 3/23) (from BD Biosciences). In order to exclude dead cells, DAPI dye (Invitrogen) was added prior to analysis, which was performed using the LSRFortessa flow cytometer with Diva software (BD Biosciences).

### 2.7 Primary stimulation of splenic cells by transduced DCs

The ability of the modified DCs to activate primary antigen-specific immune response was evaluated in a 5-day co-culture of mature DCs (0.18×10^6^ cells) and naive spleen cells (1.8×10^6^ cells) in the presence of recombinant human (rh)IL−2 (200 U/ml, Immunotools). In order to determine the percentage of CD107a^+^ cells, the degranulation assay was performed. Primarily stimulated spleen cells were incubated for 2 hours with MC38 cells in the presence of monoclonal anti−CD107a antibodies conjugated with APC (clone 1D4B, BioLegend) together with ionomycin (1 µg/ml, Sigma-Aldrich), phorbol-12-myristate-13-acetate (50 ng/ml, Sigma-Aldrich) and rhIL-2 (200 U/ml). Afterwards, cells were harvested and stained with anti−CD45 Brilliant Violet 605 (clone 30-F11), anti−CD4 FITC (clone RM4-5), anti−CD8a APC-Fire (clone 53-6.7) and anti−NK1.1 PE-Dazzle 594 (clone PK136) (all from BioLegend). For the elimination of the dead cells, DAPI dye was used. The flow cytometry analysis was performed using the LSRFortessa flow cytometer with Diva software (BD Biosciences).

### 2.8 Therapeutic treatment schedule

Eight-to-ten-week-old female C57BL/6 mice were subcutaneously (s.c.) inoculated in the right flank with MC38 cells (1.1×10^6^ cells/0.2 ml NaCl 0.9%/mouse). When tumor nodules were palpable, mice were randomly divided on the basis of tumor volume into ten or eleven experimental groups in immuno- and chemoimmunotherapeutic schedule, respectively. In both experiments, cellular vaccines were inoculated peritumorally (p.t.) on the 17^th^, 24^th^ and 31^st^ days of the experiment (2×10^6^ cells/0.2 ml NaCl 0.9%/mouse/injection). The anti-IL-10R antibodies (BioXCell) at a dose of 250 µg/0.2 ml/mouse/injection were administered intraperitoneally (i.p.) the day before injection of the cellular vaccines (16^th^, 23^rd^ and 30^th^ day of the experiment). The therapeutic schedule of chemoimmunotherapy was started on the 14^th^ day of the experiment with intravenously (i.v.) HES-MTX nanoconjugate injection in the tail vein (at a dose 20 mg/kg body weight). The HES-MTX preparation was described in detail in our previous papers (10, 12). In both experiments, three types of cellular vaccines were used – non-transduced DC/TAg, DC transduced with a control vector (DC/Vctrl/TAg) and DCs modified to overproduce cytokines. Mice received 2×10^6^ modified dendritic cells per 1 injection, regardless of whether the mice received cells overproducing only one cytokine or a mixture of cells producing two or three cytokines. Cellular vaccines named DC/IL-12/TAg, DC/IL-15/IL-15Rα/TAg and DC/IL-18/TAg consisted of 2×10^6^ cells of appropriate transductants, DC/IL-12/TAg + DC/IL-15/IL-15Rα/TAg, DC/IL-12/TAg + DC/IL-18/TAg and DC/IL-15/IL-15Rα/TAg + DC/IL-18/TAg consisted of 1×10^6^ cells of each listed transductants, while DC/IL-12/TAg + DC/IL-15/IL-15Rα/TAg + DC/IL-18/TAg was a mixture of three different transductants in the number of 0.667×10^6^ cells each.

### 2.9 Tumor volume of tumor-bearing mice

During the experiment, two or three times a week, tumors were measured by using an electronic caliper and tumor volume was calculated according to the formula: $a/2\times b^{2}$ , where represents the largest and represents the smallest tumor diameter (38). MC38 tumor growth is shown as relative tumor volume. Relative tumor volume was defined as the ratio of tumor volume on the day of measurement to that tumor volume on the day of randomization (day 13). Tumor growth kinetics are presented as a non-linear least squares regression fits of the Gompertz function. The therapeutic efficacy was determined on the basis of the tumor growth inhibition (TGI) value calculated as follows: $TGI\;(\%)=100-(TV_{t}/TV_{nt}\times 100)$ , where $TV_{t}$ refers to a median tumor volume in the treated group and *TV_nt_* – median tumor volume in the non-treated (nt) group (37). Seven days after the last DC-based vaccine injection, spleen and tumor nodules were dissected from MC38-tumor bearing mice, homogenized, and stored in liquid nitrogen for further *ex vivo* analyses.

### 2.10 Analysis of myeloid and lymphoid cell populations in MC38 tumors after applied therapy

Single-cell suspensions of tumor tissue were thawed and stained for identification of myeloid and lymphoid cell subpopulations. Tumor suspensions were stained with the LIVE/DEAD Fixable Violet Dead Staining Kit (Thermo Fisher Scientific, Inc.) and then labeled with cocktails of fluorochrome−conjugated monoclonal antibodies: anti−CD3 PE−CF594 (clone 145-2C11), anti−CD19 PE−CF594 (clone 1D3), anti−NK1.1 PE−Dazzle 594 (clone PK136) (all from BD Biosciences), anti−CD45 Brilliant Violet 605 (clone 30-F11), anti−CD11b PerCP−Cy5.5 (clone M1/70), anti−CD11c Brilliant Violet 650 (clone N418), anti−F4/80 Alexa Fluor 700 (clone BM8), anti−Ly6C PE (clone HK1.4), anti−Ly6G APC−Cy7 (clone 1A8), anti−MHC II FITC (clone M5/114.15.2), (all from BioLegend) for myeloid cell identification, and anti−CD45 Brilliant Violet 605 (clone 30-F11), anti−CD3 Brilliant Violet 650 (clone 17A2), anti−CD4 FITC (clone RM4-5), anti−CD8 APC/Fire 750 (clone 53-6.7), anti-CD19 Alexa Fluor 700 (clone 6D5), anti−CD25 PE (clone PC61) (all from BioLegend) for lymphocyte identification. Then, cells were fixed using the Foxp3/Transcription Factor Staining Buffer Set (eBioscience). Cells stained with myeloid, or lymphocyte cocktail were additionally incubated with anti−CD206 APC (clone C068C2) (BioLegend) or anti−FoxP3 APC (clone FJK-16s) (eBioscience) monoclonal antibodies, respectively. The flow cytometry analysis was performed using the LSRFortessa flow cytometer with Diva software (BD Biosciences).

### 2.11 Analysis of the systemic antitumor immune response of spleen cells after therapy

Spleen single-cell suspensions (2×10^6^ cells) were thawed and cocultured with mitomycin C-treated MC38 cells (0.1×10^6^ cells) in the presence of rhIL−2 (200 U/ml). After 5 days of restimulation, cells were harvested and the cytotoxic activity of effector splenocytes against target (MC38) cells stained with DiO lipophilic dye (Molecular Probes) was analyzed according to the previously described procedure (38). Two E:T (effector to target) ratios were investigated: 10:1 and 30:1. The dead target cells were distinguished with propidium iodide (PI, Life Technologies) solution and the percentage of DiO^+^PI^+^ MC38 cells was determined. The degranulation assay was used as described earlier (section: *Primary stimulation of splenic cells by transduced dendritic cells*). Supernatants were collected and stored at 4°C until ELISA was performed.

### 2.12 Measurement of cytokine production

Production of cytokines was evaluated using commercially available ELISA kits (IL−10, IL−4; BD Biosciences and IL-12, IL-15/IL-15Rα, IL-18, IFN−γ; eBioscience) according to the manufacturer’s instructions.

### 2.13 Statistical analyses

All data were analyzed using GraphPad Prism 9 software (GraphPad Software, Inc.). The normality of the residuals was confirmed by the D’Agostino−Pearson omnibus test. When data were consistent with a Gaussian distribution and had equal SD values, the statistical significance was calculated using the parametric one−way ANOVA followed by Tukey’s multiple comparison *post−hoc* test. When data were consistent with a Gaussian distribution, but SD values were not equal, the Brown−Forsythe and Welch ANOVA test followed by Dunnett’s T3 multiple comparisons *post−hoc* test was performed. Data inconsistent with a Gaussian distribution were analyzed using the nonparametric Kruskal−Wallis test followed by Dunn’s multiple comparison *post−hoc* test. The statistical significance of tumor growth kinetics was calculated using the two−way ANOVA followed by Tukey’s multiple comparisons *post−hoc* test. The type of statistical analysis used is described in the captions under the figures. All statistically significant differences are presented in the graphs in the form of symbols described in **Table 1**.

**Table 1:** Statistical significance markings on graphs.

p value	wording	determination of statistical significance in relation to the group:			other
		non-treated control (nt)	HES-MTX 20 mg/kg bw (H-M)	DC/Vctrl/TAg	
< 0.0001	extremely significant	****	####	xxxx	**** above the line
0.0001 to 0.001	extremely significant	***	###	xxx	*** above the line
0.001 to 0.01	very significant	**	##	xx	** above the line
0.01 to 0.05	significant	*	#	x	* above the line
≥ 0.05	not significant	no check mark	no check mark	no check mark	no check mark

## 3 Results

### 3.1 Characteristics of genetically modified DC-based vaccines

**Figure 1:**
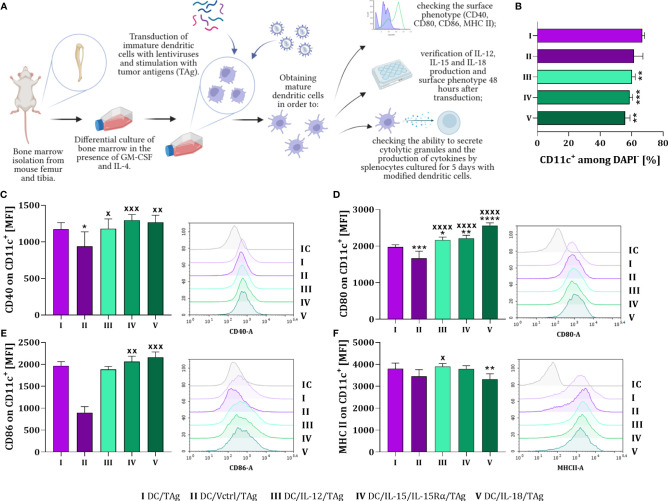
The influence of lentiviral transduction with *il12*, *il15* and *il15Rα* or *il18* genes on the phenotype of bone marrow-derived dendritic cells (DCs). **(A)** Preparation schedule of genetically modified DCs. Cells were isolated from the long bones of mice and cultured for 6 days in the presence of GM-CSF and IL-4. On day 7^th^, immature dendritic cells were transduced with cytokine genes and then stimulated with tumor antigens. On the 8^th^ day of culture, the level of differentiation of the developed vaccine cells was determined. Preparation schedule created with BioRender.com. **(B)** Percentage of CD11c^+^ cells on the 8^th^ day of DC cultures. Expression of costimulatory molecules **(C)** CD40, **(D)** CD80, **(E)** CD86 and **(F)** MHC II on the surface of CD11c^+^ cells. Results are presented as mean+SD calculated for 5-6 samples per group. Differences between groups were estimated using the non-parametric Kruskal-Wallis test followed by Dunn’s multiple comparisons *post-hoc* test **(E)**, the parametric one-way ANOVA followed by Tukey’s multiple comparisons *post-hoc* test **(C, D, F)** or the parametric Brown-Forsythe and Welch ANOVA test followed by Dunnett’s T3 multiple comparisons *post-hoc* test **(B)**. The asterisks (*) presented in the graphs indicate statistically significant differences between the given groups and the DC/TAg control cells; crosses (X) indicate a statistically significant difference between the given group and the DC/Vctrl/TAg control cells – (*/^x^p<0.05; **/^xx^p<0.01; ***/^xxx^p<0.001; ****/^xxxx^p<0.0001). IC – *isotype control*.

In the first stage of our research, we obtained mature bone marrow-derived dendritic cells (DCs) capable of increased production of interleukin: IL-12, IL-15 or IL-18 (DC/IL-12/TAg, DC/IL-15/IL-15Rα/TAg; DC/IL-18/TAg). The appropriate gene for each cytokine was introduced by transduction with lentiviral vectors. Dendritic cells were stimulated with a tumor lysate (tumor antigens, TAg) and harvested on the 8^th^ day of culture to determine their phenotypic and functional characteristics. DCs transduced with the control vector (DC/Vctrl/TAg) or non-transduced DCs (DC/TAg) stimulated with TAg were used as a control (**Figure 1A**). We observed a decrease in the percentage of CD11c^+^DAPI^-^ cells after transduction with vectors carrying genes of the particular cytokines in relation to the DC/TAg group (**Figure 1B**).

Phenotype analysis showed that the type of introduced gene considerably affected the maturation of DCs. The expression of CD40, CD80, CD86 costimulatory and MHC II molecules was determined on the surface of these cells (**Figures 1C–F**). DCs modified to cytokine production showed increased expression of CD40 and CD80 molecules compared to cells transduced with the control vector (DC/Vctrl/TAg) (**Figures 1C, D**). Compare to that control, a significantly increased expression of CD86 molecules was noticed after DC transduction with *il15* and *il18* genes (**Figure 1E**). Whereas only slight differences in the expression of MHC II molecules on the surface of these cells were observed (**Figure 1F**). The highest expression of MHC II was found on the surface of DC/IL-12/TAg and the lowest on DC/IL-18/TAg.

**Figure 2:**
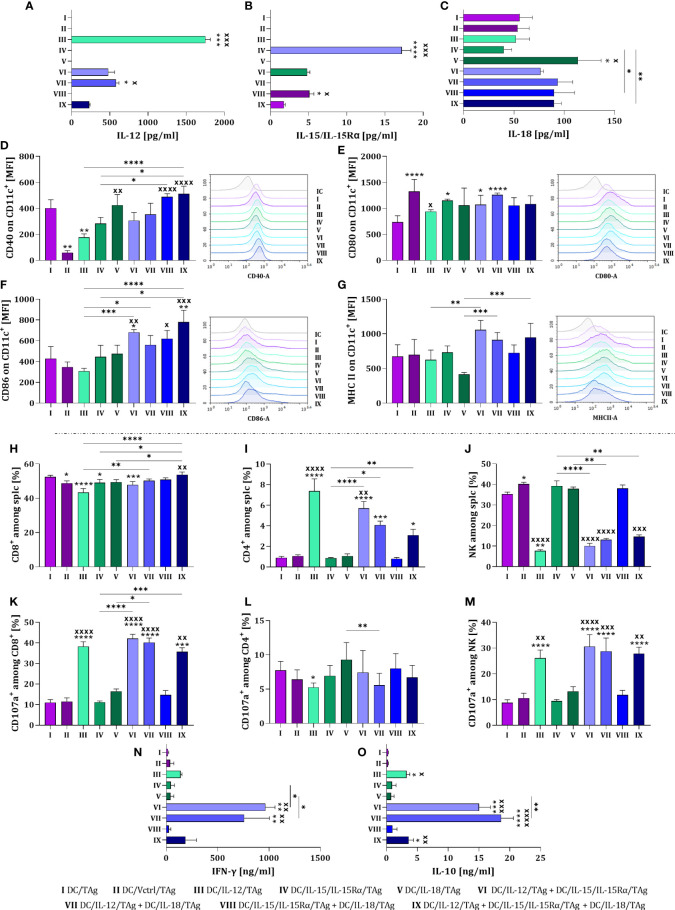
Estimation of the transduction efficiency of dendritic cells with lentiviral vectors carrying sequences of *il12*, *il15* and *il15rα* or *il18* genes. Concentration of **(A)** IL-12, **(B)** IL-15/IL-15Rα and **(C)** IL-18 in supernatants collected on the 10^th^ day of DC culture was measured using ELISA. Expression of costimulatory molecules **(D)** CD40, **(E)** CD80, **(F)** CD86 and **(G)** MHC II on the surface of CD11c^+^ cells on the 10^th^ day of DC culture measured by flow cytometry. On the 8^th^ day of the culture, dendritic cells and splenocytes were co-cultured for 5 days. After this time, splenocyte activity after primary stimulation with genetically modified DCs was determined. Percentage of **(H)** CD8^+^, **(I)** CD4^+^, and **(J)** NK cells among activated splenocytes. Percentage of CD107a^+^ cells among **(K)** CD8^+^, **(L)** CD4^+^ and **(M)** NK cells. Concentration of **(N)** IFN-γ and **(O)** IL-10 in supernatants after co-cultured activated spleen cells with modified DCs, measured using ELISA assay. Results are presented as mean+SD calculated for 6-12 samples per group. Differences between groups were estimated using the non-parametric Kruskal-Wallis test followed by Dunn’s multiple comparisons *post-hoc* test **(A–O)**. The asterisks (*) presented in the graphs indicate statistically significant differences between the given groups and the DC/TAg control cells; crosses (X) indicate a statistically significant difference between the given group and the DC/Vctrl/TAg control cells; asterisks (*) under the line indicate statistically significant differences between the given groups – (*/^x^p<0.05; **/^xx^p<0.01; ***/^xxx^p<0.001; ****/^xxxx^p<0.0001). IC, *isotype control*.

To assess the mutual influence of each overproduced cytokine on the level of DCs differentiation and activation of the antitumor response, all DC types were cultured for 48 hours in different configurations. We tested the simultaneous effect of two (DC/IL-12/TAg + DC/IL-15/IL-15Rα/TAg; DC/IL-12/TAg + IL-18/TAg and DC/IL-15/IL-15Rα/TAg + DC/IL-18/TAg) or three (DC/IL-12/TAg + DC/IL-15/IL-15Rα/TAg + DC/IL-18/TAg) cytokines on the DC phenotype and induction of a specific cellular response (**Figure 2**). Based on our former experience with using DCs as anticancer vaccines, we have known that one of the essential parameters affecting the effectiveness of the therapy is the number of injected cells. Therefore, to determine the effect of DC modifications on their antitumor activity we decided to apply the same number of cells to every single injection regardless of the composition of the vaccines. Thus, we tested control vaccines (DC/TAg and DC/Vctrl/TAg) and vaccines consisting of DCs producing one cytokine – one-component vaccine (DC/IL-12/TAg, DC/IL-15/IL-15Rα/TAg, DC/IL-18/TAg), a 1:1 mixture of two types of DCs – two-component vaccine (DC/IL-12/TAg + DC/IL-15/IL-15Rα/TAg; DC/IL-12/TAg + IL-18/TAg and DC/IL-15/IL-15Rα/TAg + DC/IL-18/TAg), or a 1:1:1 mixture of three types of DCs – three-component vaccine (DC/IL-12/TAg + DC/IL-15/IL-15Rα/TAg + DC/IL-18/TAg).

The highest concentration of introduced cytokines was noted in cultures with DCs producing one cytokine (groups III, IV and V) (**Figures 2A–C**). In two-component or three-component vaccines, a reduced concentration of interleukins was observed, which was related to the number of individual cytokine-producing cells in the mixture. Despite this, in mixed cultures (groups VI-IX), a greater influence of cytokines on changes in the phenotype of DCs than in monocultures was observed. The highest expression of the CD40 molecule was demonstrated on the surface of DC/IL-18/TAg cells and in a mixed culture of these cells with DC/IL-15/IL-15Rα/TAg and DC/IL-12/TAg + DC/IL-15/IL-15Rα/TAg (**Figure 2D**). No effect of cytokines on changes in the expression of the CD80 on the surface of vaccine cells was observed (**Figure 2E**). However, a slight increase in the expression of this molecule was shown in the culture of transduced DCs compared to DC/TAg. This may suggest that the presence of CD80 on the surface of transduced DCs is related to viral rather than cytokine stimulation. The expression of CD86 molecules was increased in the DCs mixed cultures, especially in relation to cells transduced with the control vector (**Figure 2F**). However, such an effect was not observed on the surface of one-component vaccines. An increase in the level of MHC II expression on transduced DCs in mixed cultures compared to the monoculture was determined (**Figure 2G**).

The DC ability to primary stimulate naïve T cells and induce a specific antitumor response was assessed in a 5-day co-culture of splenocytes with all types of vaccine cells. Changes in the percentage of CD8^+^, CD4^+^ and NK cells, as well as their ability for degranulation assessed by the expression of the CD107a molecule on their surface, were determined. Moreover, we investigated the ability of stimulated splenocytes to produce IFN-γ and IL-10.

The largest population of splenocytes in co-cultures were CD8^+^ cells, which accounted for approx. 50% of all spleen cells stimulated with DCs. An increase in the percentage of these cells was observed when splenocytes were cultured with the three-component vaccine in relation to co-culture with one-component vaccines and cells modified with the control vector (**Figure 2H**). A decisive effect of DC/IL-12/TAg or their mixture with other transductants was found to increase the percentage of CD4^+^ cells in the co-culture (**Figure 2I**), and oppositely – a decrease in NK cell percentage was revealed (**Figure 2J**). Among the CD8^+^ cell populations, the highest expression of the CD107a molecule was observed after splenocytes contact with DCs overproducing IL-12, regardless of whether it was produced alone or in combination with other cytokines (**Figure 2K**). No significant differences in the size of the CD107a cell population were observed among CD4^+^ cells (**Figure 2L**), while among NK cells (**Figure 2M**) a statistically significant increase was observed when splenocytes were co-cultured with DCs overproducing IL-12 (groups III, VI, VII and IX). Although increased production of IFN-γ (**Figure 2N**) and IL-10 (**Figure 2O**) was detected in all groups with DC/IL-12/TAg, the highest concentration of these cytokines was observed after splenocyte stimulation with DC/IL-12/TAg and DCs overproducing IL-15 or IL-18 (groups VI and VII).

The tested cellular vaccines were capable of secreting cytokines whose genes have been introduced using a third-generation lentiviral system. Although cellular vaccines consisting of dendritic cell mixtures produced correspondingly smaller amounts of particular cytokines, the effect of these combinations revealed stronger stimulation of DCs. Thus, a preliminary assessment of the effectiveness of DCs transduced with cytokine genes and stimulated with TAg showed their significant effect on inducing a specific cellular response in *ex vivo* conditions. Dendritic cells overproducing IL-12 alone or in combination with other cytokines could activate CD4^+^, CD8^+^, and NK cells, and consequently, increase their ability to produce IFN-γ and IL-10. This indicates that this way prepared vaccines can be potent inducers of the anticancer immune response.

### 3.2 The influence of DC-based vaccines on tumor growth inhibition

In the next research stage, we determined whether, similarly to *in vitro* conditions, the combination of transductants will cause a better therapeutic effect manifesting the inhibition of tumor growth than one-component vaccines administered in the same final number of cells. In immunotherapeutic protocol, we decided to precede the administration of cell-based vaccines with an intraperitoneal injection of anti-IL-10R antibodies to make the immune system cells insensitive to the adverse effects of IL-10 produced in TME. This decision was based on our previous *in vitro* observations that vaccine dendritic cells are able to activate the splenocytes to produce the high IFN-γ amount accompanied by IL-10 secretion. Furthermore, after completing immunotherapy with cellular vaccines supported with anti-IL-10R antibodies, we conducted another chemoimmunotherapy experiment – in which the previous immunotherapy was supplemented with the administration of the HES-MTX nanoconjugate.

**Figure 3:**
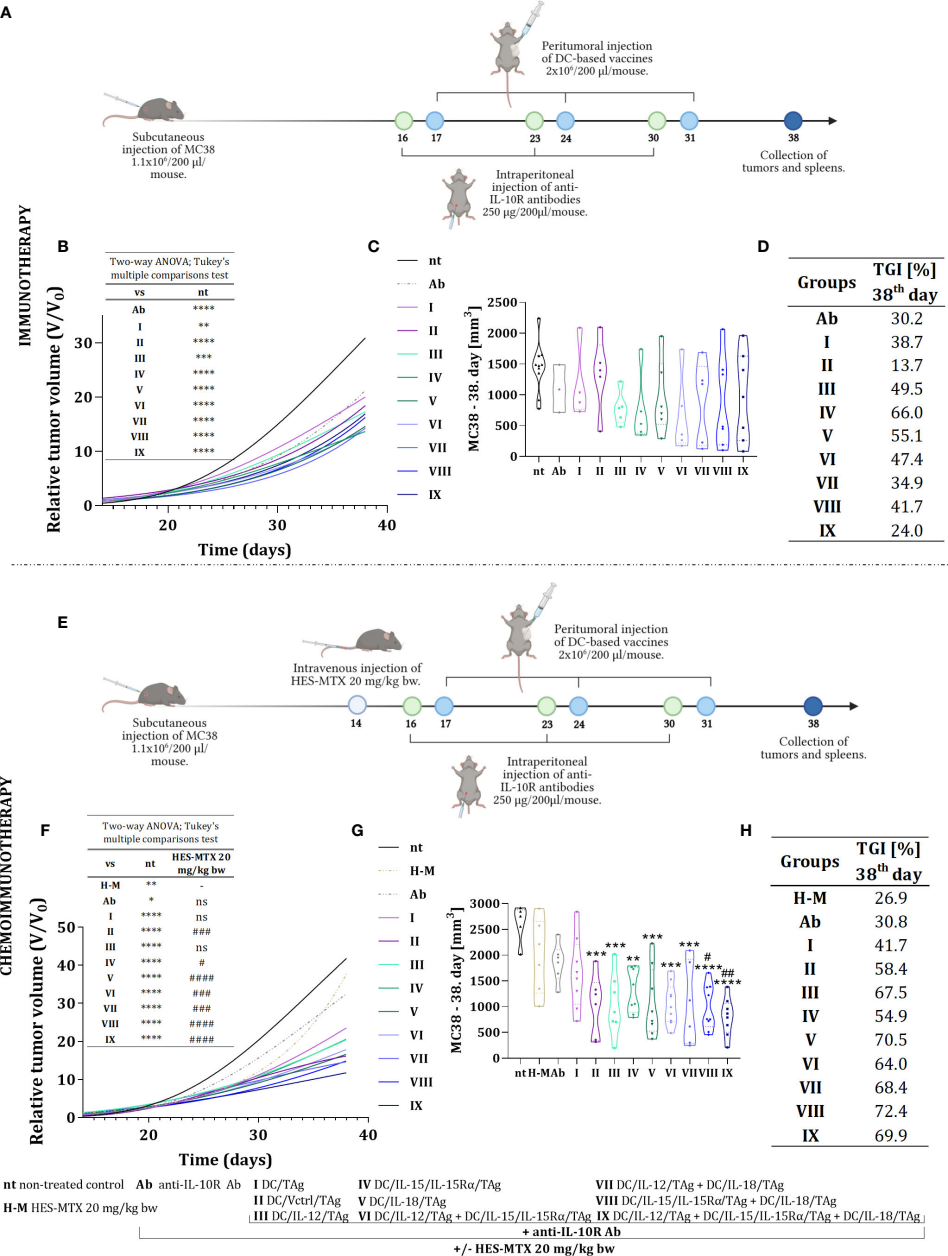
Growth of MC38 tumors in mice treated with immunotherapy or chemoimmunotherapy with nanoconjugate HES-MTX followed by multiple injections of anti-IL-10R antibodies and DC-based vaccines. Treatment schedule of immunotherapy **(A)** and chemoimmunotherapy **(E)** created with BioRender.com. **(B, F)** Graph presenting the growth kinetics of MC38 tumor (shown as relative tumor volume) in mice after immunotherapy or chemoimmunotherapy (data was normalized). **(C, G)** Truncated violin plot presenting individual tumors volume and designated median tumor volume for each group, calculated on the 38^th^ day of the immunotherapy or chemoimmunotherapy experiment. Results are presented as median for 3-10 mice per group. **(D, H)** Table presenting MC38 tumor growth inhibition (TGI) calculated on the 38^th^ day of the experiment in relation to the non-treated group (nt). Differences between groups were estimated using the two−way ANOVA followed by Tukey’s multiple comparisons *post−hoc* test **(B, E)** or nonparametric Kruskal−Wallis test followed by Dunn’s multiple comparisons test **(C, G)**. The asterisks (*) presented in the graphs indicate statistically significant differences between the given groups and the non-treated control group (nt); hashtags (#) above a bar indicate a statistically significant difference between the given group and the HES-MTX treated group (H-M) – (*/^#^p<0.05; **/^##^p<0.01; ***/^###^p<0.001; ****/^####^p<0.0001).

On the 16^th^, 23^rd^, and 30^th^ day, mice were inoculated intraperitoneally with anti-IL-10R antibodies (250 µg/mouse). Day after (17^th^, 24^th^, and 31^st^ day), cellular vaccines were administered peritumorally (p.t.) (2×10^6^ cells/mice). As control cells, DC/TAg, and DC/Vctrl/TAg were used. On the 38^th^ day, the therapeutic effect of the treatment was determined (**Figure 3A**). In the chemoimmunotherapy experiment, the protocol was supplemented with an intravenous injection of HES-MTX (20 mg/kg b.w.) on the 14^th^ day of the experiment (**Figure 3E**).

Based on tumor volume measurements carried out during the therapeutic experiments, tumor growth curves (**Figures 3B, F**), and violin plots (**Figures 3C, G**) were prepared to determine the kinetics of tumor growth. The efficacy of both therapies was estimated based on relative tumor volume - the ratio of tumor volume on the day of measurement to tumor volume on the day of randomization (day 13). This method of presentation depicted the tumor growth rate relative to their initial volume and unified the differences between individual therapeutic groups. Hence, on the 38^th^ day of the experiment, the degree of tumor growth inhibition (TGI) in the therapeutic groups compared to the non-treated group was calculated (**Figures 3D, H**).

The use of anti-IL10R antibodies in therapy inhibited the tumor growth rate in relation to the group of non-treated mice (TGI 30.2%). Moreover, the application of anti-IL10R antibodies and DC-based vaccines influenced the changes in tumor growth rate and this effect depended on the type of applied vaccine. It should be underlined that immunotherapy with one-component vaccines resulted in the greatest inhibition of tumor growth rate compared to the non-treated (nt) group (**Figure 3D**). The MC38 tumors growth inhibition in these groups ranged from 49.5% (for DC/IL-12/TAg) to 66% (for DC/IL-15/IL-15Rα/TAg). The effect of the two-component vaccines on TGI was weaker and an after the application of anti-IL-10R antibodies and the three-component vaccine, an ineffective treatment was shown.

In our previous studies, we found that using 20 mg/kg b.w. of the HES-MTX nanoconjugate leads to beneficial immunomodulation of the antitumor response (12). Therefore, this chemotherapeutic agent was administered to enhance the therapeutic effect of the herein used immunotherapy. The extension of the therapeutic scheme with the use of the HES-MTX prior to immunotherapy resulted in a strong increase in the value of TGI. The combined therapy resulted in the greatest inhibition of tumor growth when the vaccine containing DC/IL-15/IL-15Rα/TAg and DC/IL-18/TAg cells (72.4%, group VIII) was applied, whereas the use of the one-component vaccine (DC/IL-15/IL-15Rα/TAg or DC/IL-18/TAg, group IV and V, respectively) induced tumor growth inhibition on the level of 54.9% and 70.5% respectively (**Figure 3H**). Nevertheless, the most surprising result of combined chemoimmunotherapy was obtained after the administration of the three-component vaccine - supplementing the treatment schedule with the HES-MTX nanoconjugate administration resulted in an increase of TGI value to 69.9% in contrast to 24.0%, which was observed in this group but in the immunotherapeutic scheme of treatment.

Thus, our observations can suggest that the effect of the applied immunotherapy depends not only on the amount of delivered cytokines but also on their combination. Nonetheless, the administration of the HES-MTX nanoconjugate facilitated the enhancement of vaccine efficacy, even when the amount of produced cytokines turned out too weak to contribute to the therapeutic effect in the course of immunotherapy.

### 3.3 Leukocyte infiltration of tumor nodules in applied therapies

**Figure 4:**
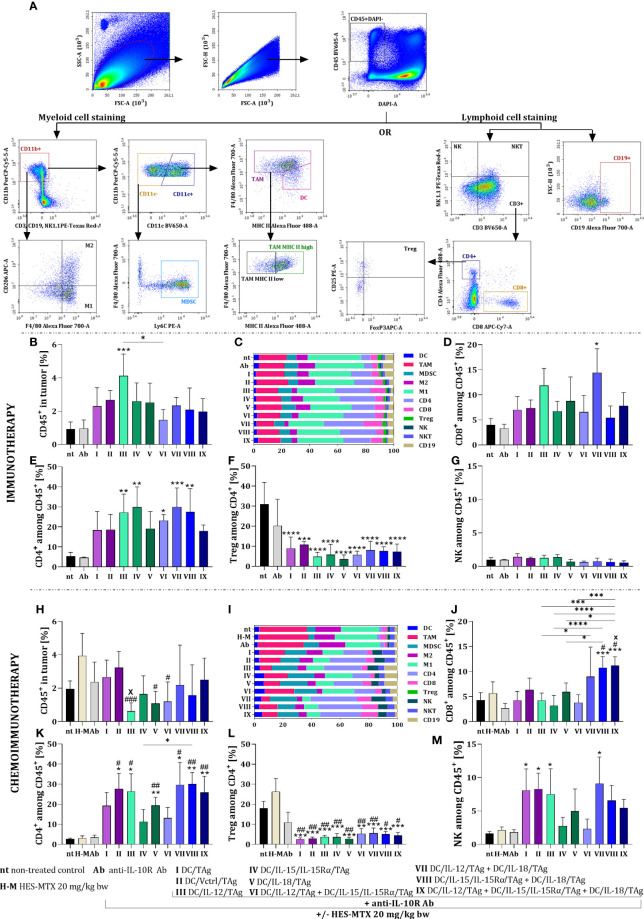
Influence of applied immunotherapy or chemoimmunotherapy on MC38 tumor nodules infiltration with leukocytes. **(A)** Scheme of the multiparameter flow cytometric analysis of lymphoid and myeloid cells in tumor tissue. **(B, H)** Percentage of live CD45^+^ cells in tumor tissue. Percentage of **(C, I)** each leukocyte population, **(D, J)** CD8^+^, **(E, K)** CD4^+^, **(G, M)** NK cells among CD45^+^ cells in tumor. **(F, L)** Percentage of T regulatory lymphocytes among CD4^+^ cells in tumor tissue. Results are presented as mean+SD calculated for 3-7 mice per group. Differences between groups were estimated using the non-parametric Kruskal-Wallis test followed by Dunn’s multiple comparisons *post-hoc* test **(J, M)**, the parametric one-way ANOVA followed by Tukey’s multiple comparisons *post-hoc* test **(B, D, H, K, L)** or the parametric Brown-Forsythe and Welch ANOVA test followed by Dunnett’s T3 multiple comparisons *post-hoc* test **(E, F)**. The asterisks (*) presented in the graphs indicate statistically significant differences between the given groups and the non-treated control group (nt); hashtags (#) above a bar indicate a statistically significant difference between the given group and the HES-MTX treated group (H-M); crosses (X) indicate a statistically significant difference between the given group and DC/Vctrl/TAg treated group; asterisks (*) under the line indicate statistically significant differences between the given groups – (*/^#^/^x^p<0.05; **/^##^p<0.01; ***/^###^p<0.001; ****p<0.0001).

Interested in the changes in the tumor growth rate between the applied therapies, we decided to check their influence on leukocyte infiltration into tumors. For this purpose, MC38 tumor nodules were harvested on the 38^th^ day of therapy and multiparameter cytometric analyses were performed to identify myeloid and lymphoid cell influxes (**Figure 4A**). Briefly, among alive leukocytes (CD45^+^DAPI^-^), we determined the effect of the applied therapies on changes in the percentage of lymphoid cells among leukocytes such as lymphocytes T CD8 (CD3^+^CD8^+^), T CD4 (CD3^+^CD4^+^), Treg (CD3^+^CD4^+^CD25^+^FoxP3^+^), NK (CD3^-^NK1.1^+^), NKT (CD3^+^NK1.1^+^) and B lymphocytes (CD19^+^). There were also distinguished populations of the myeloid cells (CD11b^+^) including DCs (CD11c^+^F4/80^int^MHC II^+^), TAMs (CD11c^+^F4/80^+^), MDSCs (CD11c^-^Ly6C^+^), M1 (CD206^-^) and M2 (CD206^+^) macrophages. Despite extensive cytometric analysis of cells infiltrating tumor tissue, we did not observe significant differences in all populations. Therefore we decided to discuss only some of them in more detail.

After the application of immunotherapy, we observed a slight increase in the percentage of leukocytes in tumor tissue obtained from mice treated with DC-based vaccines, which was the highest when DC/IL-12/TAg was used. (**Figure 4B**). However, based on summary graphs presenting normalized data, we also visualized the differences in individual subpopulations of leukocytes infiltrating tumor tissue (**Figure 4C**).

The percentage of CD8^+^ cells infiltrating tumor tissue increased after the administration of cellular vaccines and anti-IL10R antibodies. However, a statistically significant increase was observed only after the use of DC/IL-12/TAg + DC/IL-18/TAg (**Figure 4D**). Application of DC-based vaccines generated an increase in the percentage of CD4^+^ cells relative to the non-treated (nt) and antibodies-treated (Ab) groups. A statistically significant increase in the percentage of CD4^+^ cells was observed after the use of DC/IL-12/TAg and DC/IL-15/IL-15Rα/TAg and two-component vaccines (**Figure 4E**). At the same time, there was a substantial reduction in the population size of regulatory T cells among CD4^+^ cells in tumor tissue. This decrease in the percentage of Tregs was rather related to the use of DC-based vaccines, regardless of the ability of DCs to overproduce cytokines (**Figure 4F**). We also observed a slight reduction in the percentage of NK cells after the administration of DC-based vaccines consisted of more than one type of produced cytokines (groups VI-IX) (**Figure 4G**).

After chemoimmunotherapy, a slight reduction in the leukocyte population size was noted in all therapeutic groups (**Figure 4H**). The lowest percentage of CD45^+^ cells was found after using DC/IL-12/TAg (group III). In addition, there were substantial differences between the effects of different types of vaccines on TAM, M1, M2, CD4+, CD8+, and NK cell population sizes (**Figure 4I**).

A statistically significant increase in the percentage of CD8^+^ cells was observed as a result of chemoimmunotherapy containing a mixture of DCs overproducing IL-15 and IL-18 (group VIII) or all three tested cytokines (group IX) in relation to control groups (groups nt, H-M) and one-component DC-based vaccines. Furthermore, a slight increase in the size of the CD8^+^ cell population was observed after the administration of DC/IL-12/TAg + DC/IL-18/TAg (group VII) (**Figure 4J**). The high percentage of CD4^+^ cells in tumor tissue was always observed when DC-based vaccines were applied (**Figure 4K**). In both T lymphocyte subpopulations, using the nanoconjugate prior to immunotherapy was the most conducive to the effect of two or three-component vaccines. The administration of the chemotherapeutic agent did not enhance the effect of the immunotherapy on changes in the size of Treg cell population (**Figure 4L**). Meanwhile, an increase in the percentage of NK cell population was visible, especially in groups I, II, III and VII (**Figure 4M**).

**Figure 5:**
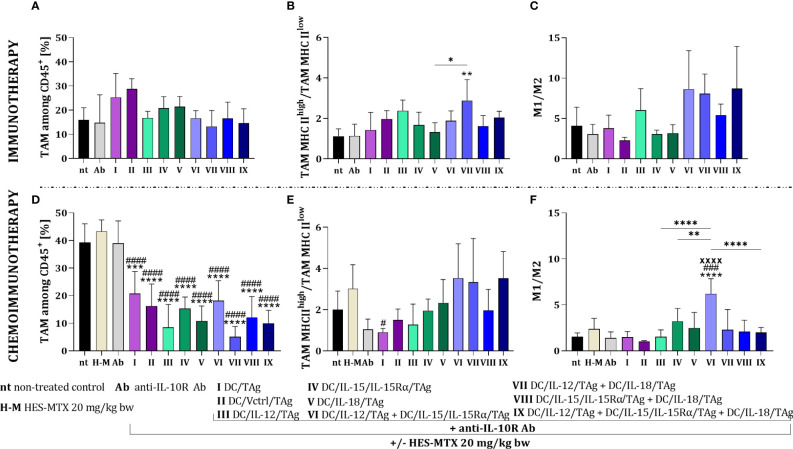
Evaluation of macrophage polarization in MC38 tumor tissue after immunotherapy or chemoimmunotherapy. Scheme of the multiparameter flow cytometric analysis of macrophage subpopulation in MC38 tumor nodules obtained from mice after immunotherapy or chemoimmunotherapy is shown in **Figure 4A**. **(A, D)** Percentage of TAM among CD45^+^ cells in tumors. **(B, E)** TAM MHC II^high^/TAM MHC II^low^ and **(C, F)** M1/M2 ratios showing the polarization of TAMs in MC38 tumor tissue. Results are presented as mean+SD calculated for 3-7 mice per group. Differences between groups were estimated using the non-parametric Kruskal-Wallis test followed by Dunn’s multiple comparisons *post-hoc* test **(E)** or the parametric one-way ANOVA followed by Tukey’s multiple comparisons *post-hoc* test **(B, D, F)**. The asterisks (*) presented in the graphs indicate statistically significant differences between the given groups and the non-treated control group (nt); hashtags (#) above a bar indicate a statistically significant difference between the given group and the HES-MTX treated group (H-M); crosses (X) indicate a statistically significant difference between the given group and the DC/Vctrl/TAg treated group; asterisks (*) under the line indicate statistically significant differences between the given groups – (*/^#^p<0.05; **p<0.01; ***/^###^p<0.001; ****/^####^/^XXXX^p<0.0001).

In the case of the immunotherapeutic treatment schedule, the applied DC-based vaccines did not cause significant changes in the percentage of myeloid cells in tumor tissue. Although there was no effect of this therapy on changes in the size of the TAM population (**Figure 5A**), we observed differences in their activation level. Based on the ratio of TAM MHC II^high^ to TAM MHC II^low^, we found that in the groups receiving cellular vaccines, TAMs with high expression of MHC II were slightly dominated and the highest value of this ratio was observed after the administration of a two-component DC vaccine containing DC/IL-12/TAg and DC/IL-18/TAg (**Figure 5B**). Besides, analyzing the effect of therapy influences on macrophage influx, we observed an increased M1 to M2 macrophage ratio in the groups receiving two-component and three-component DC vaccines (**Figure 5C**).

The combined therapy with the use of the HES-MTX nanoconjugate induced statistically significant changes in the TAM percentage relative to the non-treated or the chemotherapeutic treated groups (**Figure 5D**). The highest value of the TAM MHC II^high^/TAM MHC II^low^ ratio was shown after the administration of chemoimmunotherapy containing two or three-component vaccines comprising DC/IL-12/TAg (**Figure 5E**). On the other hand, the highest value of the M1/M2 ratio was demonstrated after therapy with HES-MTX, anti-IL-10R and DC/IL-12/TAg + DC/IL-15/IL-15Rα/TAg (**Figure 5F**).

The use of the HES-MTX nanoconjugate before immunotherapy created optimal conditions for the effective action of cell-based vaccines. The most favorable effect was observed in the groups receiving dendritic cell mixtures, especially the three-component vaccine.

### 3.4 Influence of multicomponent treatment on systemic antitumor response

**Figure 6:**
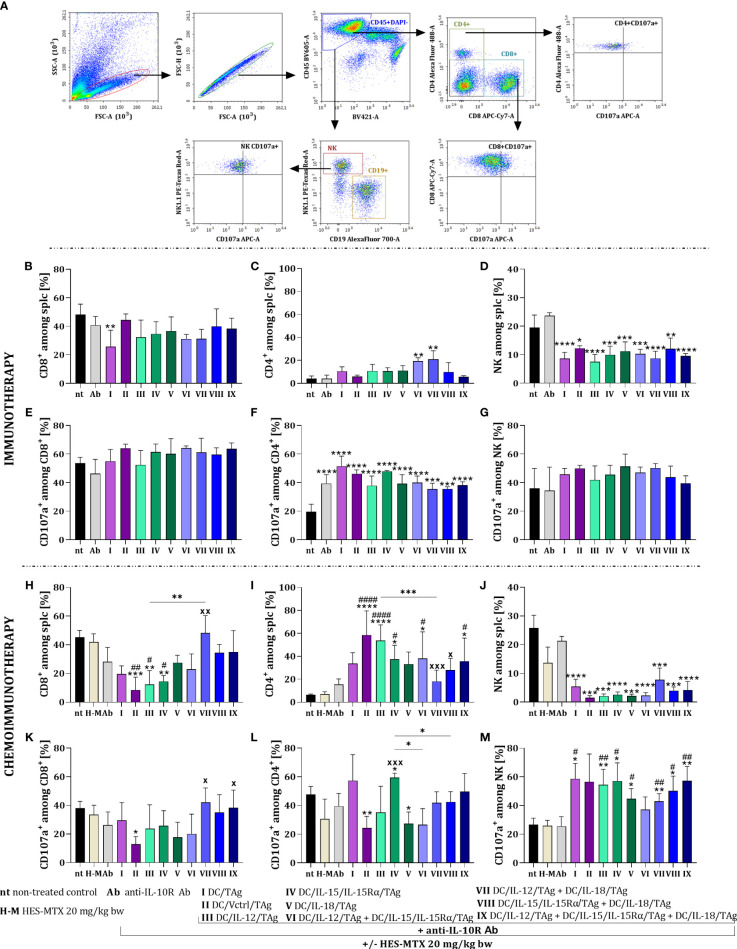
Effect of applied immunotherapy and chemoimmunotherapy on activation of the restimulated splenocytes. **(A)** Scheme of the multiparameter flow cytometric analysis of activated CD8^+^, CD4^+^, and NK cells among restimulated splenocytes measured by CD107a degranulation assay. Percentage of **(B, H)** CD8^+^, **(C, I)** CD4^+^, and **(D, J)** NK cells among restimulated splenocytes. Percentage of CD107a^+^ cells among **(E, K)** CD8^+^, **(F, L)** CD4^+^ and **(G, M)** NK cells. Results are presented as mean+SD calculated for 3-7 mice per group. Differences between groups were estimated using the non-parametric Kruskal-Wallis test followed by Dunn’s multiple comparisons *post-hoc* test **(C)**, the parametric one-way ANOVA followed by Tukey’s multiple comparisons *post-hoc* test **(B, D, F, H–M)**. The asterisks (*) presented in the graphs indicate statistically significant differences between the given groups and the non-treated control group (nt); hashtags (#) above a bar indicate a statistically significant difference between the given group and the HES-MTX treated group (H-M); crosses (X) indicate a statistically significant difference between the given group and the DC/Vctrl/TAg treated group; asterisks (*) under the line indicate statistically significant differences between the given groups – (*/^#^/^x^p<0.05; **/^##^/^xx^p<0.01; ***/^xxx^p<0.001; ****/^####^p<0.0001).

To confirm the assumption that the enhancement of the effect of both multicomponent immunotherapy and chemoimmunotherapy depends mainly on the use of DCs mixtures, we assessed their effect on the activity of systemic antitumor response. For this purpose, on the 38^th^ day of both therapies’ spleens from treated tumor-bearing mice were harvested and restimulated with MC38 cells in a 5-day mixed culture. Next, multiparameter cytometric analyses were performed among alive splenocytes (CD45^+^DAPI^-^), to identify changes in the percentage of CD8^+^, CD4^+^ and NK cells as well as their ability to secrete cytolytic granules based on the expression of the CD107a molecule (**Figure 6A**).

There was no significant change in the size of the CD8^+^ cell population among restimulated splenocytes obtained from mice after immunotherapy with cytokine-secreting DC-based vaccines (**Figure 6B**). Solely after the application of anti-IL-10R and DC/TAg (group I) the percentage of these cells was significantly lower than in the untreated group. An increase in the percentage of CD4^+^ cells was seen after treatment with cellular vaccines, especially after administration of DC/IL-12/TAg + DC/IL-15/IL-15Rα/TAg and DC/IL-12/TAg + DC/IL-18/TAg (**Figure 6C**). However, after the application of all cell-based vaccines, there was a reduction in the percentage of NK cell population (**Figure 6D**). The applied therapy did not significantly change the percentage of CD107a^+^ cells among CD8^+^ cells (**Figure 6E**) and NK cells (**Figure 6G**) in comparison to their controls. Meanwhile, the ability to release cytolytic granules increased among CD4^+^ cell populations, turn to be related to the anti-IL-10R antibodies administration (**Figure 6F**).

The supplementation of treatment with the HES-MTX nanoconjugate caused a reduction in the percentage of CD8^+^ cells, especially after DC/Vctrl/TAg (group II), DC/IL-12/TAg (group III), and DC/IL-15/IL-15Rα/TAg (group IV). However, the visible increase in the percentage of this population in relation to DC/Vctrl/TAg occurred after the administration of DC/IL-12/TAg + DC/IL-18/TAg (group VII) vaccines (**Figure 6H**). The administration of combined therapy with cytostatic caused an increase in the percentage of CD4^+^ cells, like immunotherapy, but the changes concerned other therapeutic groups. This cell population’s size increased after the DC/Vctrl/TAg (group II) and DC/IL-12/TAg (group III) vaccines. An enhanced effect of DC/IL-15/IL-15Rα/TAg and the three-component vaccine on the population of restimulated splenic CD4^+^ cells (**Figure 6I**) was also observed. The administration of the chemotherapeutic agent and DC-based vaccines supported with anti-IL-10R antibodies deepened the reduction in the percentage of NK cells in relation to immunotherapy alone (**Figure 6J**). The effect of the applied chemoimmunotherapy on the size of the cell subpopulations able to release cytolytic granules was also revealed. The lowest ability to secrete cytolytic granules was found in CD8^+^ cells obtained from mice treated with HES-MTX + anti-IL-10R + DC/Vctrl/TAg (**Figure 6K**). In contrast, among CD4^+^ cells, the highest cytolytic activity was observed in the group of mice receiving dendritic cells modified to overproduce IL-15/IL-15Rα (**Figure 6L**). The size of NK CD107a^+^ population increased in all groups receiving DC-based vaccines in relation to control groups (groups: nt, H-M and Ab) (**Figure 6M**).

Comparison of both therapies harnessing schedules, including treatment with antibodies, and/or additional supplementation with HES-MTX, resulted in marked differences compared to the non-treated group. The use of cytostatic agent deepened variations among particular groups and enhanced the mixture effect of the DC-based vaccines, especially these three-component. Consequently, such a multicomponent combination enhanced the systemic activity of main populations of immune cells.

**Figure 7:**
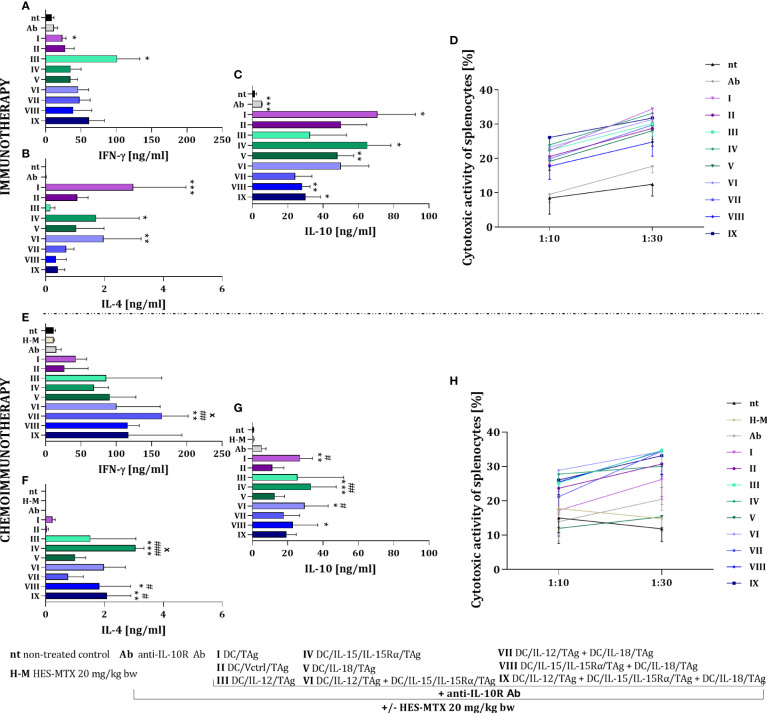
Impact of conducted immunotherapy and chemoimmunotherapy on activation of the systemic antitumor response. Concentration of **(A, E)** IFN-γ, **(B, F)** IL-4 and **(C, G)** IL-10 in supernatants after restimulation of spleen cells with MC38 cells, measured using ELISA assay. **(D, H)** Cytotoxic activity of splenocytes (effector cells, E) against DiO^+^ MC38 cells (target cells, T) after 4-hour incubation in 10:1 and 30:1 E:T ratios, measured using flow cytometry. Results are presented as mean+SD calculated for 3-7 mice per group. Differences between groups were estimated using the non-parametric Kruskal-Wallis test followed by Dunn’s multiple comparisons *post-hoc* test **(B, C, E–G)** or the parametric Brown-Forsythe and Welch ANOVA test followed by Dunnett’s T3 multiple comparisons *post-hoc* test **(A)**. The asterisks (*) presented in the graphs indicate statistically significant differences between the given groups and the non-treated control group (nt); hashtags (#) above a bar indicate a statistically significant difference between the given group and the HES-MTX treated group (H-M); crosses (X) indicate a statistically significant difference between the given group and the DC/Vctrl/TAg treated group – (*/^#^/^x^p<0.05; **/^##^p<0.01; ***/^###^p<0.001).

As confirmation of the potential for antitumor activity, the ability of restimulated splenocytes to produce cytokines and their cytotoxic activity against MC38 cells was examined. In the case of immunotherapy, splenocytes obtained from mice treated with DC/IL-12/TAg were able to produce the highest amount of IFN-γ (**Figure 7A**). The highest production of IL-4 was noted after the application of the therapy consisted of DC/IL-15/IL-15Rα/TAg (group IV) and DC/IL-12/TAg + DC/IL-15/IL-15Rα/TAg (group VI) (**Figure 7B**). In contrast, increased production of IL-10 by splenocytes was shown in the groups of mice treated with DC-based vaccines, as well as anti-IL-10R antibodies alone (**Figure 7C**). The highest cytotoxic activity of splenocytes against MC38 cells (**Figure 7D**) was observed in all groups treated with DC-based vaccines, especially after the application of DC/TAg (group I), DC/IL-15/IL-15/Rα/TAg (group IV) and the three-component vaccine (group IX).

The use of the HES-MTX nanoconjugate prior to immunotherapy strongly changed the type and level of cytokine production by splenocytes causing an increase in their ability to produce IFN-γ (**Figure 7E**). Thus, while the production level of this cytokine did not change markedly after the administration of DC/IL-12/TAg alone, the splenocyte’s ability to produce IFN-γ increased after the use of DCs mixed cultures, especially in DC/IL-12/TAg + DC/IL-18/TAg group (group VII). The HES-MTX nanoconjugate also influenced changes in the production of IL-4 by spleen cells obtained from mice after therapy. The highest ability to produce IL-4 was characterized by splenocytes obtained from mice treated with combination therapy with DC/IL-15/IL-15Rα/TAg (group IV), DC/IL-15/IL-15Rα/TAg + DC/IL-18/TAg (group VIII) and three-component vaccine (group IX) (**Figure 7F**). Splenocytes obtained from mice which were treated with vaccines containing DCs showed a decrease in IL-10 production which suggest the influence of the HES-MTX nanoconjugate on the prolongation of the decrease of suppressor cell activity (**Figure 7G**). The addition of the nanoconjugate involved a slight increase in the splenocytes’ cytotoxic activity compared to immunotherapy. However, it depended on the type of vaccine and did not reveal which modified dendritic cell mixtures should be considered the most powerful (**Figure 7H**).

The observed changes in the systemic anticancer response confirm that the use of the HES-MTX chemotherapeutic agent has the most beneficial action on the cellular vaccines that are a mixture of two or three transductants. In these groups, an increase in IFN-γ and a decrease in IL-10 production, as well as an enhancement in the cytotoxic activity of restimulated splenocytes are particularly visible.

**Figure 8:**
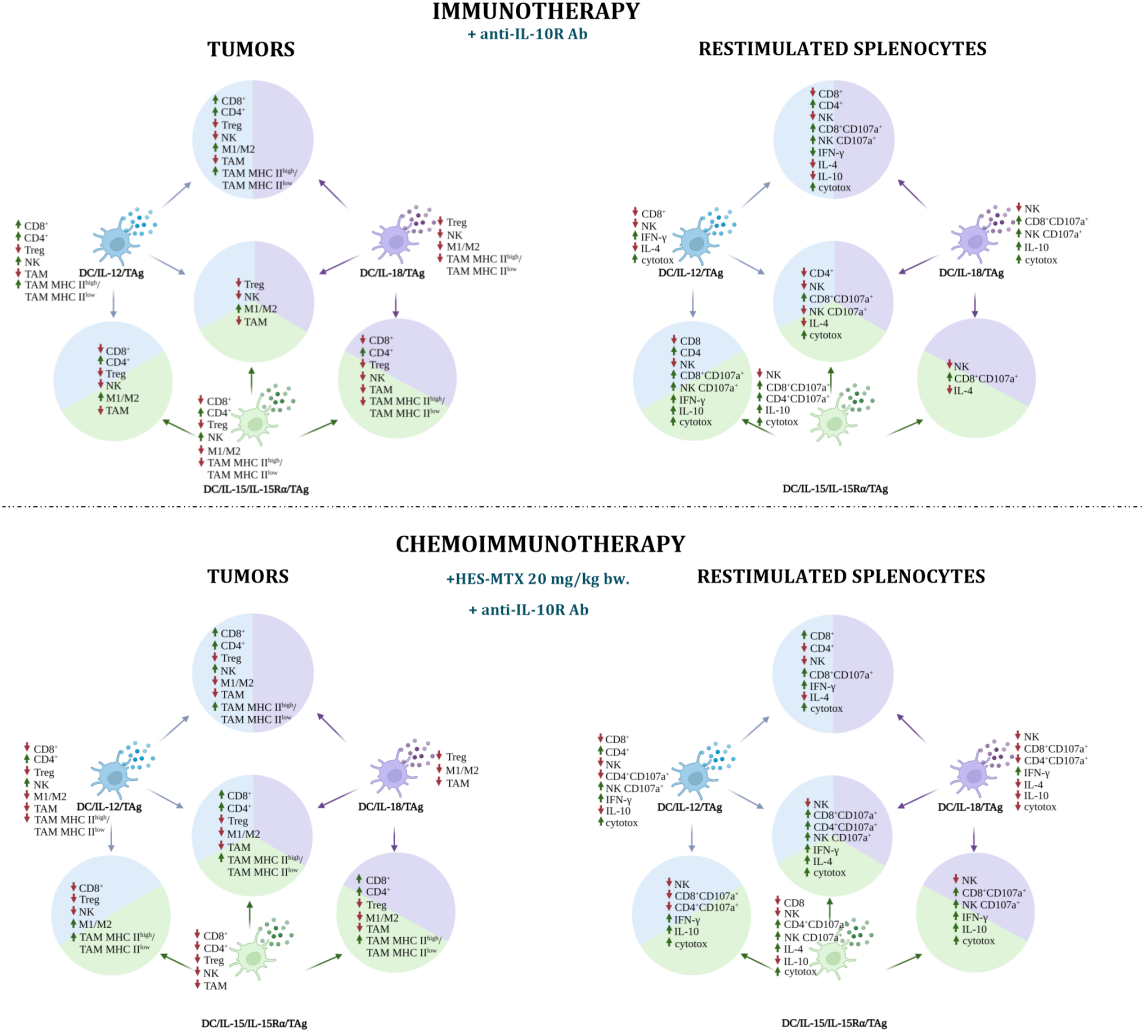
Impact of immunotherapy and chemoimmunotherapy based on DC-based vaccines on individual populations of immune cells in tumors and restimulated splenocytes. The diagrams show the effect of cellular vaccines on the increase (up arrow ↑) and decrease (down arrow ↓) of CD8^+^, CD4^+^, Treg, NK, TAM cell populations and M1/M2 and TAM MHC II^high^/TAM MHC II^low^ ratios in tumors and of CD8^+^, CD4^+^, NK, CD107a^+^ cell populations, IFN-γ, IL-10 production and cytotoxic activity among restimulated splenocytes. The figure was created with BioRender.com.

The use of the developed vaccines in anticancer therapy in combination with anti-IL-10R antibodies and/or a chemotherapeutic agent influenced both the changes in the tumor microenvironment and the activation of the systemic immune response. Both the antibodies and the chemotherapeutic administered prior to DC-based vaccines created a favorable microenvironment in which the vaccines were characterized by different mechanisms of action (**Figure 8** and **Supplementary Tables 1**–**4**) mainly representing the effect of overproduced cytokines. By virtue of the use of each of the developed vaccines, the percentage of Treg lymphocytes in both immuno- and chemoimmunotherapy decreased. The supplement of the cellular vaccines treatment with the HES-MTX nanoconjugate affected the enhanced tumor growth inhibition, with the exception of the DC/IL-15/IL-15Rα/TAg.

In the immunotherapy, the DC/IL-12/TAg mainly increased the influx of CD8^+^, CD4^+^ and NK cells to tumor nodules, which was accompanied by a decrease in the population of Treg and TAMs and an increase in IFN-γ production and cytotoxic activity of splenocytes. The addition of HES-MTX agent had a positive effect primarily on the systemic immune response. The use of DC/IL-15/IL-15Rα/TAg increased the influx of CD4^+^ and NK cells into tumors. The potential of splenocytes to release cytolytic granules by CD8^+^ and CD4^+^ cells, IL-10 production and cytotoxic activity also increased. Meanwhile, supplementation of the immunotherapy schedule with nanoconjugate did not favorably affect the effect of this vaccine in tumor tissue, resulting in a reduction of CD4^+^ and NK cell percentage. In addition, a decrease in the percentage of CD8^+^ cells and no change in the cytotoxic activity of this cell subpopulation among the restimulated splenocytes was observed. However, this treatment resulted in an increase in the percentage of CD4^+^CD107a^+^, NK CD107a^+^ cells, IL-4 production and cytotoxic activity. Dendritic cells overproducing IL-18 mainly affected the percentage reduction of Treg lymphocytes, NK cells, M1/M2 and TAM MHC II^high^/TAM MHC II^low^ ratios in tumors, while the population of CD8^+^CD107a^+^ and NK CD107a^+^ cells and cytotoxic activity of splenocytes increased. The therapy combined with nanoconjugate caused the reduction of TAM percentage. Although among the restimulated splenocytes the percentage of cells capable of releasing cytolytic granules and production of IL-10 decreased, the production of IFN-γ increased.

In immunotherapy, all two-component vaccines caused a reduction of TAMs and NK cell populations in tumors, but a higher percentage of CD8^+^CD107a^+^ cells was observed among restimulated splenocytes. During immunotherapy, the DC/IL-12/TAg + DC/IL-15/IL-15Rα/TAg vaccine increased the percentage of CD4^+^ cells and the M1/M2 macrophages ratio in tumors, while the percentage of CD8^+^ cells, Treg, NK and TAMs decreased. Among the splenocytes, a higher percentage of CD4^+^, CD8^+^CD107a^+^ and NK CD107a^+^ cells, production of IFN-γ and IL-10 and cytotoxic activity were observed. During chemoimmunotherapy, only the M1/M2 ratio remained at an increased level and a high TAM MHC II^high^/TAM MHC II^low^ ratio was observed. Among splenocytes, in addition to a decrease in the population of NK, CD8^+^CD107a^+^, CD4^+^CD107a^+^cells, an increase in the production of IFN-γ, IL-10 and cytotoxic activity was revealed. Administration of DC/IL-12/TAg + DC/IL-18/TAg resulted in an augmented influx of CD4^+^ and CD8^+^ cells into the tumors and growth of M1/M2 and TAM MHC II^high^/TAM MHC II^low^ ratios. Among the splenocytes, an increase in the percentage of CD4^+^ cells and cytolytic activity of CD8^+^ and NK cells was observed, as well as higher production of IFN-γ and a decrease in the production of both IL-4 and IL-10, which could have enhanced the cytotoxic activity of splenocytes. The addition of the chemotherapeutic intensified the influx of NK cells, but the M1/M2 ratio decreased. In the spleens, an increase in the percentage of CD8^+^ cells was observed, with a simultaneous reduction of CD4^+^ cell percentage. During immunotherapy, a vaccine combining DC/IL-15/IL-15Rα/TAg and DC/IL-18/TAg induced tumor growth inhibition of 41.7%, which may be the result of the influx of CD4^+^ cells with a simultaneous reduction in the percentage of tumor suppressor Tregs and TAMs. However, this vaccine induced a decrease in the percentage of CD8^+^ and NK cells. Among the splenocytes, a decrease in the percentage of NK cells and a higher percentage of CD8^+^CD107a^+^ cells were observed. The addition of the chemotherapeutic agent resulted in this vaccine eliciting the highest tumor growth inhibition (72.4%), which may have been due to the additional influx of CD8 lymphocytes and the increase in the TAM MHC II^high^ to TAM MHC II^low^ ratio, while the overall TAM cell population, Treg, M1/M2 ratio decreased. In restimulated splenocytes, an increased percentage of CD8^+^ and NK cells capable of releasing cytolytic granules was observed, and in addition, these cells were capable of high IFN-γ and IL-10 production and were characterized by high cytotoxic activity.

The three-component vaccine DC/IL-12/TAg + DC/IL-15/IL-15Rα/TAg + DC/IL-18/TAg during immunotherapy showed a negligible therapeutic effect. Inhibition of tumor growth was only 24%, and we did not observe an increased influx of CD4^+^ and CD8^+^ cells in tumors, but a higher M1/M2 macrophage ratio and a decrease in the percentage of Treg, NK cells and TAMs. In the spleens, a reduction of CD4^+^, NK, NK CD107a^+^ cell percentage and IL-4 production was revealed, while an increase in the percentage of CD8^+^CD107a^+^ cells and cytotoxic activity was observed. However, the addition of the HES-MTX nanoconjugate to the therapy resulted in enhanced tumor inhibition (69.9%), an augmented influx of CD8^+^, CD4^+^ cells, and a higher TAM MHC II^high^/TAM MHC II^low^ ratio in a tumor, at the same time reducing the percentage of Treg, M1/M2 ratio and TAMs. On the other hand, in the spleens, we observed an increase in the cytolytic activity of CD4^+^, CD8^+^ and NK cells, an increase in the production of IFN-γ and IL-4 and cytotoxic activity.

## 4 Discussion

The main aim of our study was to determine whether the administration of HES-MTX before immunotherapy would change the antitumor effect of dendritic cells modified for overproduction of IL-12, IL-15, or IL-18. As numerous literature reports showed a beneficial effect resulting from the combination of different cytokines we decided to apply a combination of modified cells (39–41). Furthermore, DC-based therapy was enriched with components able to reduce the hostile impact of TME on applied vaccines. For this purpose, we used antibodies directed against the IL-10R receptor, which should partially block the negative effect of IL-10 on the cells of the immune system (23, 42) and HES-MTX as an immunomodulatory chemotherapeutic agent.

We started our research by characterizing the obtained dendritic cell transductants. Modification of DCs with a lentiviral control vector (DC/Vctrl/TAg) caused a short-term reduction in the expression of costimulatory molecules CD40, CD80, CD86, and MHC II, which is consistent with the research of French scientists (43). However, cytokines’ action abolished the transduction’s negative effect. Compared to monocultures, the increased expression of costimulatory molecules and MHC II was observed on the two-component cellular vaccines. Nevertheless, three-component vaccines revealed the highest maturity despite the lowest production of single cytokines. It showed that the cooperation of these cytokines might potentiate DC activity, which confirmed reports, that the presence of proinflammatory cytokines in the environment of DCs allows for achieving a higher degree of cell maturity than after activation with TAg alone (37). Many works showed the influence of IL-12, IL-15, or IL-18 on changes in the surface phenotype of dendritic cells (44–47). However, none of them tested the effect of these three cytokines simultaneously.

As part of the functional characterization of the vaccine cells, the ability of transduced TAg-stimulated DCs to activate splenocytes was assessed. Among the splenocytes from co-culture, changes in the percentage of CD4^+^, CD8^+^, NK, and CD107a^+^ cells were determined, as well as the production of IFN-γ and IL-10. The CD8^+^ and NK cells cultured in the presence of DCs modified to overproduce IL-12 were characterized by the greatest ability to secrete cytolytic granules, regardless of whether DCs were used as a monoculture or in combination with other cytokines. Notably, the activation level of CD8^+^ and NK cells was similar in each of these mixtures. However, splenocytes cultured with DC/IL-12/TAg + DC/IL-15/IL-15Rα/TAg or DC/IL-12/TAg + DC/IL-18/TAg had the highest potential for IFN-γ secretion. This observation is consistent with numerous studies showing the synergistic effect of IL-12 and IL-18 or IL-15 on IFN-γ production (48–50). Moreover, Martinović et al. showed that simultaneous stimulation by IL-12 and IL-18 induced an increase in the cytotoxic activity of NK cells and expression of CD107a on their surface, as well as IFN-γ production, than independent stimulation (51).

We used the tested cellular vaccines in two experiments - immunotherapeutic and chemoimmunotherapeutic. In the immunotherapeutic part, antibodies blocking the IL-10 receptor were administered to mice prior to the application of cellular vaccines. This was conducted due to reports that in cancer patients elevated IL-10 levels are associated with a poorer prognostic (52, 53). Moreover, Llopiz et al. observed that IL-10 induced in DCs less mature phenotype and decreased T-cell activation capacity (54). Therefore, it seems justified to use antibodies blocking the IL-10 receptor to abolish the negative impact of this cytokine on all immune cells.

The applied therapies based on dendritic cells caused an increase in the percentage of CD4^+^ and CD8^+^ cells among cells infiltrating tumor tissue and a simultaneous decrease in the percentage of Treg cells among CD4^+^ lymphocytes. Although literature data indicate the influence of IL-12, IL-15 and IL-18 on the increase in the percentage of NK cells (55), we did not observe such changes in our studies. It should be emphasized that in the data presented by Oka et al. study recombinant cytokines were used in high doses. In our research, dendritic cells were used as producers of these cytokines, which released them over a long period, but in lower concentrations.

The use of the HES-MTX nanoconjugate before administration of the immunotherapy enhanced the therapeutic efficacy of the vaccines, especially those three-component. In the case of immunotherapy alone, the use of a three-component vaccine did not significantly inhibit tumor growth (24.0% TGI), while the addition of cytostatic affected its effectiveness (69.9% TGI). It should be highlighted that the three-component vaccine showed the highest anti-cancer potential in *in vitro* studies. This may indicate that ability of these types of DC-based vaccines to trigger effective anti-tumor immune response has been diminished by a hostile tumor microenvironment. HES-MTX alone resulted in a 26.9% inhibition of tumor growth. As literature has shown, other methotrexate-based chemotherapeutics, such as glucose-methotrexate conjugate (GLU-MTX) and hydroxyethyl cellulose-methotrexate (HEC-MTX) also caused significant tumor growth delay *in vivo* in breast cancer-bearing mice (56, 57).

The inclusion in the therapy of a chemotherapeutic agent further reduced the percentage of TAMs in the groups receiving anti-IL-10R antibodies and cellular vaccines, especially those capable of releasing cytokines. The reduction of TAMs is a good prognostic marker because these cells can promote tumor progression by producing factors and cytokines that support tumor cell proliferation (58). The decrease in the TAM percentage observed after chemoimmunotherapeutic treatment should be related to the immunomodulatory potential of the HES-MTX, which we have already confirmed in our previous work (12). We had shown, that on the third day after the HES-MTX administration, the percentages of cells with suppressor activity (such as TAMs, Tregs) had decreased while the infiltration of CD8^+^ and NK cells into MC38 tumor tissue had increased. Thus, we postulate, that application of the HES-MTX created the optimal tumor milieu for DCs applied p.t., which were capable of generating an efficient antitumor immune response in such an environment. In turn, this contributed to the further decrease in the TAM percentage and an increased influx of effector lymphocytes (CD8^+^, NK cells) into MC38 tumor tissue. In addition, the level of MHC II expression may indicate changes in tumor progression. The population of TAM MHC II^high^ is associated with the early phase of tumor development and tumor suppression, whereas TAM MHC class II^low^ dominates as the tumor progresses (5). Our research showed an increase in the TAM MHC II^high^/TAM MHC II^low^ ratio caused primarily by the effect of cell-based vaccines, especially two- or three-component vaccines.

In particular, it should be emphasized that the supplementation of immunotherapy with the administration of the HES-MTX significantly influenced the activation of the systemic anticancer response. The use of the chemotherapeutic or anti-IL-10R antibodies alone did not affect the degree of activation of restimulated splenocytes. However, the use of two- or three-component vaccines secreting, among others, IL-12, increased the percentage of cells capable of releasing cytolytic granules, compared to the vaccine based on dendritic cells modified with the control vector. The highest cytotoxic activity was found in restimulated splenocytes obtained from mice that received immuno- and chemoimmunotherapy based on two- and three-component vaccines, however, chemoimmunotherapy induced increased production of IFN-γ.

To sum up, the use of the HES-MTX nanoconjugate prior to immunotherapy involving multiple administrations of anti-IL-10R antibodies and DC-based vaccines capable of overproducing proinflammatory cytokines IL-12, IL-15 or IL-18 created optimal conditions for the effective action of these vaccines in mouse colorectal cancer. Of these, two- or three-component vaccines revealed the greatest potential for use in anticancer therapy characterized by the highest level of expression of costimulatory molecules and MHC II. The applied chemoimmunotherapy caused the highest inhibition of tumor growth in the group receiving DC/IL-15/IL-15Rα/TAg + DC/IL-18/TAg at the level of 72.4%. But HES-MTX also enhanced the activity of the three-component vaccine leading to 69.9% inhibition of tumor growth in course of chemoimmunotherapy, compared to 24.0% in immunotherapy alone. A decrease in the percentage of Treg cells was observed in both applied therapeutic schedules. Apart from this, chemoimmunotherapy using DC-based vaccines induced an increase in NK cell infiltration into the tumor and a decrease in the percentage of TAM. Nevertheless, the highest ratio of TAM MHC II^high^ to TAM MHC II^low^ was noticed when mice received two- or three-component vaccines. The use of dendritic cells as vaccines increased cytotoxic activity in both experiments, but the highest ability to both kill tumor cells and produce IFN-γ was found in splenocytes obtained from mice receiving two- or three-component vaccines in chemoimmunotherapy.

## 5 Conclusions

In chemoimmunotherapy, two- or three-component vaccines had the greatest potential, even though individual cytokines were produced in smaller amounts than in a single-component vaccine. Their use resulted in the greatest inhibition of tumor growth and effective response of immune cells. The obtained results suggest that the developed chemoimmunotherapy may have a promising application in anticancer therapy.

## Data availability statement

The raw data supporting the conclusions of this article will be made available by the authors, without undue reservation.

## Ethics statement

The animal study was reviewed and approved by Local Ethic Committee for Experiments with the Use of Laboratory Animals, Wrocław, Poland (authorization number 077/2019).

## Author contributions

Conceptualization, KW-C and EP-P; Methodology, EP-P, JR, TG.; Formal Analysis, KW-C, JM, AS and EP-P; Investigation, KW-C, JM, AS, JR, AW, MŚ, BS-O and EP-P; Resources, EP-P; Supervision, EP-P; Writing – Original Draft Preparation, KW-C and EP-P; Writing – Review and Editing, KW-C, EP-P, JM, AS, AW, JR, BS-O, TG, MŚ; Project Administration, KW-C and EP-P; Funding Acquisition, EPP. All authors have read and agreed to the published version of the manuscript.
