# Supplementary material for: Inhibition of MC38 colon cancer growth by multicomponent chemoimmunotherapy with anti-IL-10R antibodies, HES-MTX nanoconjugate, depends on application of IL-12, IL-15 or IL-18 secreting dendritic cell vaccines
Source: Front Immunol. 2023 Jul 20;14:1212606. doi: 10.3389/fimmu.2023.1212606 (PMC10399586; doi:10.3389/fimmu.2023.1212606)
Supplement: Supplementary file 1 [file Table_1.docx]

Supplementary Material

Inhibition of MC38 colon cancer growth by multicomponent chemoimmunotherapy with anti-IL-10R antibodies, HES-MTX nanoconjugate, depends on application of IL-12, IL-15 or IL-18 secreting dendritic cell vaccines

**Katarzyna Węgierek-Ciura*, Jagoda Mierzejewska, Agnieszka Szczygieł, Joanna Rossowska, Anna Wróblewska, Marta Świtalska, Tomasz M. Goszczyński, Bożena Szermer-Olearnik, Elżbieta Pajtasz-Piasecka**

*** Correspondence:** Corresponding Author: katarzyna.wegierek@hirszfeld.pl

# Supplementary Data

**Supplementary Table 1.** Summary of leukocyte infiltration into tumors during immunotherapy shown as averaged values.

|  | **CD8^+^ among CD45^+^** | **CD4^+^ among CD45^+^** | **Treg among CD4^+^** | **NK among CD45^+^** | **M1/M2** | **TAM among CD45^+^** | **TAM MHC II^high^/ TAM MHC II^low^** |
| --- | --- | --- | --- | --- | --- | --- | --- |
| **nt** | 4,0 | 5,4 | 31,0 | 1,0 | 4,1 | 16,0 | 1,1 |
| **Ab anti-IL-10R** | 3,3 | 4,7 | 20,2 | 1,0 | 3,0 | 14,8 | 1,1 |
| **DC/TAg** | 7,0 | 18,4 | 8,9 | 1,4 | 3,8 | 25,3 | 1,4 |
| **DC/Vctrl/TAg** | 7,4 | 18,7 | 10,8 | 1,2 | 2,3 | 28,8 | 2,0 |
| **DC/IL-12/TAg** | 11,9 | 27,2 | 4,8 | 1,3 | 6,0 | 16,8 | 2,4 |
| **DC/IL-15/TAg** | 6,7 | 29,9 | 5,8 | 1,4 | 3,0 | 20,8 | 1,7 |
| **DC/IL-18/TAg** | 8,8 | 19,2 | 3,5 | 0,7 | 3,2 | 21,4 | 1,3 |
| **DC/IL-12/TAg + DC/IL-15/TAg** | 6,6 | 23,2 | 5,7 | 0,6 | 8,6 | 16,7 | 1,9 |
| **DC/IL-12/TAg + DC/IL-18/TAg** | 14,4 | 29,9 | 8,1 | 0,8 | 8,1 | 13,3 | 2,9 |
| **DC/IL-15/TAg + DC/IL-18/TAg** | 5,4 | 27,4 | 7,6 | 0,7 | 5,4 | 16,7 | 1,6 |
| **DC/IL-12/TAg + DC/IL-15/TAg + DC/IL-18/TAg** | 7,8 | 18,0 | 7,3 | 0,6 | 8,7 | 14,7 | 2,0 |

**Supplementary Table 2.** Summary of data from the analysis of restimulated splenocytes obtained after immunotherapy, presented as averaged values.

|  | **CD8^+^ among splc** | **CD4^+^ among splc** | **NK among splc** | **CD107a^+^ among CD8^+^** | **CD107a^+^ among CD4^+^** | **CD107a^+^ among NK** | **IFN-γ** | **IL-4** | **IL-10** | **Cytotoxic activity 1:10** | **Cytotoxic activity 1:30** |
| --- | --- | --- | --- | --- | --- | --- | --- | --- | --- | --- | --- |
| **nt** | 48,3 | 4,0 | 19,5 | 53,7 | 19,7 | 35,9 | 8,8 | 0,0 | 1,5 | 8,5 | 12,5 |
| **Ab anti-IL-10R** | 40,7 | 4,1 | 23,6 | 46,3 | 39,3 | 34,5 | 11,8 | 0,0 | 5,5 | 9,6 | 17,7 |
| **DC/TAg** | 25,7 | 10,5 | 8,6 | 54,9 | 51,5 | 45,9 | 23,8 | 3,0 | 70,9 | 22,3 | 34,5 |
| **DC/Vctrl/TAg** | 44,3 | 5,8 | 12,2 | 64,0 | 46,1 | 49,9 | 27,8 | 1,1 | 50,2 | 20,6 | 28,8 |
| **DC/IL-12/TAg** | 32,3 | 10,8 | 7,5 | 52,5 | 37,9 | 41,8 | 101,1 | 0,2 | 32,7 | 22,5 | 30,2 |
| **DC/IL-15/TAg** | 34,6 | 10,7 | 10,0 | 61,4 | 47,9 | 45,5 | 35,4 | 1,7 | 65,1 | 24,0 | 33,2 |
| **DC/IL-18/TAg** | 36,6 | 11,1 | 11,2 | 60,1 | 39,3 | 51,4 | 35,1 | 1,0 | 48,2 | 19,2 | 28,1 |
| **DC/IL-12/TAg + DC/IL-15/TAg** | 31,1 | 19,5 | 10,3 | 64,3 | 40,0 | 47,1 | 46,1 | 2,0 | 50,1 | 23,3 | 31,4 |
| **DC/IL-12/TAg + DC/IL-18/TAg** | 31,3 | 21,1 | 8,7 | 61,1 | 35,4 | 50,2 | 47,8 | 0,7 | 24,3 | 19,9 | 29,8 |
| **DC/IL-15/TAg + DC/IL-18/TAg** | 39,9 | 9,9 | 12,1 | 59,7 | 35,7 | 43,7 | 39,1 | 0,4 | 28,1 | 17,7 | 24,8 |
| **DC/IL-12/TAg + DC/IL-15/TAg + DC/IL-18/TAg** | 38,3 | 5,6 | 9,6 | 63,7 | 38,2 | 39,6 | 61,3 | 0,4 | 30,0 | 26,1 | 31,8 |

**Supplementary** **Table 3**. Summary of leukocyte infiltration into tumors during chemoimmunotherapy shown as averaged values.

|  |  |  | **CD8^+^ among CD45^+^** | **CD4^+^ among CD45^+^** | **Treg among CD4^+^** | **NK among CD45^+^** | **M1/M2** | **TAM among CD45^+^** | **TAM MHC II^high^/ TAM MHC II^low^** |
| --- | --- | --- | --- | --- | --- | --- | --- | --- | --- |
|  |  | **nt** | 3,0 | 2,5 | 24,3 | 0,7 | 1,5 | 39,3 | 2,0 |
|  |  | **HES-MTX 20 mg/kg bw** | 4,9 | 2,6 | 34,2 | 1,0 | 2,4 | 43,3 | 3,0 |
| **,+HES-MTX 20 mg/kg bw** |  | **Ab anti-IL-10R** | 1,9 | 7,5 | 13,6 | 0,8 | 1,4 | 39,0 | 1,0 |
|  | **.+Ab anti-IL-10R** | **DC/TAg** | 3,1 | 3,0 | 3,2 | 1,2 | 1,5 | 20,8 | 0,9 |
|  |  | **DC/Vctrl/TAg** | 3,7 | 1,5 | 3,5 | 1,4 | 1,0 | 16,2 | 1,5 |
|  |  | **DC/IL-12/TAg** | 2,1 | 3,9 | 4,0 | 0,8 | 1,5 | 8,4 | 1,3 |
|  |  | **DC/IL-15/TAg** | 1,5 | 8,3 | 4,5 | 0,5 | 3,2 | 15,3 | 2,0 |
|  |  | **DC/IL-18/TAg** | 3,6 | 2,8 | 3,4 | 0,5 | 2,5 | 10,8 | 2,3 |
|  |  | **DC/IL-12/TAg + DC/IL-15/TAg** | 1,9 | 1,6 | 7,2 | 0,6 | 6,2 | 18,2 | 3,5 |
|  |  | **DC/IL-12/TAg + DC/IL-18/TAg** | 7,2 | 0,5 | 5,7 | 1,4 | 2,3 | 5,1 | 3,3 |
|  |  | **DC/IL-15/TAg + DC/IL-18/TAg** | 6,5 | 1,0 | 5,2 | 1,1 | 2,1 | 12,1 | 2,0 |
|  |  | **DC/IL-12/TAg + DC/IL-15/TAg + DC/IL-18/TAg** | 8,1 | 0,7 | 5,8 | 1,6 | 2,0 | 10,0 | 3,5 |

**Supplementary** **Table 4**. Summary of data from the analysis of restimulated splenocytes obtained after chemoimmunotherapy, presented as averaged values.

|  |  |  | **CD8^+^ among splc** | **CD4^+^ among splc** | **NK among splc** | **CD107a^+^ among CD8^+^** | **CD107a^+^ among CD4^+^** | **CD107a^+^ among NK** | **IFN-γ** | **IL-4** | **IL-10** | **Cytotoxic activity 1:10** | **Cytotoxic activity 1:30** |
| --- | --- | --- | --- | --- | --- | --- | --- | --- | --- | --- | --- | --- | --- |
|  |  | **nt** | 45,2 | 6,3 | 25,9 | 38,2 | 47,7 | 26,6 | 11,6 | 0,0 | 0,7 | 15,0 | 11,8 |
|  |  | **HES-MTX 20 mg/kg bw** | 41,9 | 7,1 | 13,6 | 33,5 | 30,7 | 25,9 | 11,3 | 0,0 | 0,8 | 17,8 | 14,8 |
| **,+HES-MTX 20 mg/kg bw** |  | **Ab anti-IL-10R** | 28,2 | 15,5 | 21,3 | 26,3 | 39,6 | 25,5 | 15,1 | 0,0 | 5,4 | 14,0 | 20,5 |
|  | **.+Ab anti-IL-10R** | **DC/TAg** | 19,6 | 33,7 | 5,4 | 29,6 | 57,3 | 58,6 | 42,8 | 0,2 | 26,9 | 17,2 | 26,2 |
|  |  | **DC/Vctrl/TAg** | 8,5 | 58,5 | 1,6 | 13,0 | 24,4 | 56,6 | 26,7 | 0,0 | 1,3 | 27,7 | 30,7 |
|  |  | **DC/IL-12/TAg** | 12,4 | 53,6 | 2,1 | 23,7 | 35,1 | 54,5 | 85,9 | 1,5 | 0,5 | 22,2 | 34,7 |
|  |  | **DC/IL-15/TAg** | 14,5 | 37,4 | 2,6 | 25,7 | 59,4 | 57,1 | 68,4 | 3,1 | 2,7 | 27,8 | 30,1 |
|  |  | **DC/IL-18/TAg** | 27,5 | 33,1 | 2,2 | 17,7 | 27,4 | 44,6 | 91,1 | 1,0 | 11,6 | 12,0 | 15,4 |
|  |  | **DC/IL-12/TAg + DC/IL-15/TAg** | 23,0 | 38,0 | 2,3 | 20,1 | 26,7 | 37,2 | 100,5 | 2,0 | 48,2 | 28,9 | 34,4 |
|  |  | **DC/IL-12/TAg + DC/IL-18/TAg** | 48,1 | 18,1 | 7,8 | 42,0 | 41,9 | 42,9 | 165,0 | 0,8 | 29,4 | 21,2 | 34,4 |
|  |  | **DC/IL-15/TAg + DC/IL-18/TAg** | 34,5 | 27,9 | 3,9 | 35,0 | 42,6 | 50,2 | 115,9 | 1,8 | 43,7 | 25,4 | 34,5 |
|  |  | **DC/IL-12/TAg + DC/IL-15/TAg + DC/IL-18/TAg** | 35,0 | 35,7 | 4,3 | 38,4 | 49,7 | 57,3 | 117,2 | 2,1 | 31,9 | 26,1 | 33,2 |
